# We Eat Too Much

**Published:** 1890-09

**Authors:** 


### WE EAT TOO MUCH.

A recent writer in an English medical journal shows conclusively that, while certain classes, owing to the stress of poverty, cannot obtain the nutriment they really need, the majority of people eat too much. Fortunately a moderate degree of over eating does not appear to be markedly injurious. The digestive apparatus, though compelled to do more work than is really necessary, proves equal to the demands made upon it and does not break down or get seriously out of order. This is but one illustration out of many that might be given, showing how the marvellous mechanism of the human body adapts itself to conditions more or less abnormal. It is lucky for the average man that physiological laws are not of Medo-Persic inflexibility. He can violate them to a limited extent without incurring the penalty, though he finds that, if he goes beyond that point, the punishment is swift and sure.

Careful investigations prove that the daily " destructive metabolism," or in plain English, the inevitable waste and wear of the body, which is the measure of the *work* it does, varies but little for different occupations. A diet of from twelve to fourteen ounces of chemically dry food, if the ingredients are in proper portion and readily digestible, is sufficient to keep the average worker in good health. One part of nitrogenous to seven or eight parts of non-nitrogenous food is found to be a fair combination. A very small addition of stimulants appears to increase the amount of possible work ; but moderately free drinking diminishes it. Women eat less than men, after making allowance for differences in weight and work. Where a man eats nineteen ounces, a woman of the same weight and equally active habits eats only fourteen or fifteen ounces. This latter alllowance, as will be seen from the figures given above, is more than enough for a hard-working man, even when all meat is excluded from the diet. It is no uncommon thing, however, for a man of average size and activity to eat double this amount, or from twenty-five to twenty-seven ounces of chemically dry food in a day. We are inclined to think that excess in eating is at least no less common in this country than in England. The abundance, variety and cheapness of food are naturally favorable to this over indulgence. The palate is tempted to intemperance by appetizing dishes, when it would be fully satisfied with a normal amount of plain and wholesome food.

There are few of our readers who will not confess that the appetite when completely appeased by meat and its concomitants at dinner, is revived by the delicacies which pass under the head of dessert and are quite unnecessary. We feel that we have had enough, but the new and savory appeal to our love of the good things of the table is too much for us. We have been eating because we were hungry ; we now go on eating because we enjoy it. It is not necessary, but it is "nice." Let us congratulate ourselves that, though gluttony and intemperance are bestial sins and cannot escape their punishment, moderate over indulgence in eating is, as we have said, apparently a venial offense against the laws of health ; but let us beware of presuming upon the mercy with which Nature tempers justice in the enforcement of these laws.

---

The president of the Chicago Board of Pharmacy asserts that the women of America spend $62,000,000 a year for cosmetics, most of which have for their chief ingredients zinc, oxide, calomel, water, glycerine and perfumes.

